# Head

**Published:** 2019-11-28

**Authors:** Taryn Elaine O'Neill

**Affiliations:** 1 Department of Medicine, Dalhousie University, New Brunswick, Canada

**Figure:**
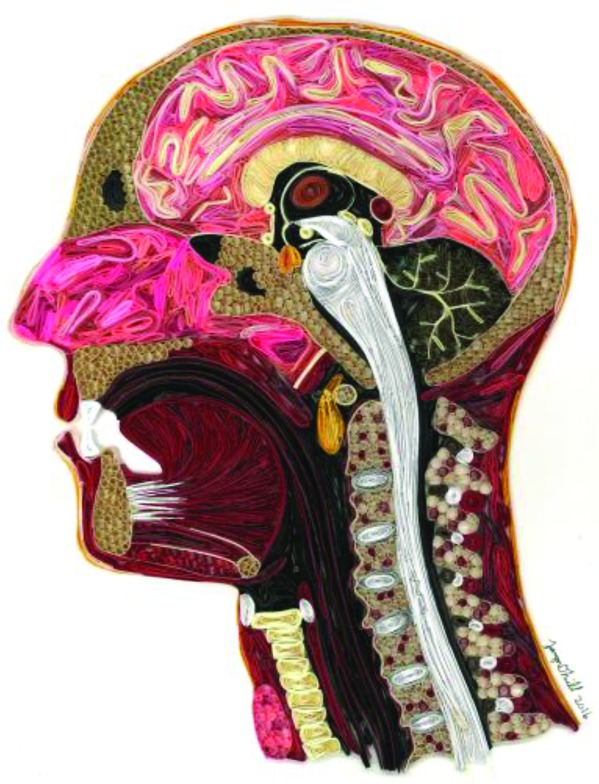


To fully understand something you must break it down to its simplest form. This is also true for medicine. In order to grasp challenging concepts, you need to have a basic comprehension of all the components, you need to see the forest before the individual trees. As a medical student I was confronted with new information and then relied on my prior learning to build a more complex and better picture.

This piece was constructed by adding small strips of paper folded in distinct patterns that as a collection form an overall image.

